# Maternal Cortisol Mediates Hypothalamus-Pituitary-Interrenal Axis Development in Zebrafish

**DOI:** 10.1038/srep22582

**Published:** 2016-03-04

**Authors:** Dinushan Nesan, Mathilakath M. Vijayan

**Affiliations:** 1 Department of Biology, University of Waterloo, Waterloo, Ontario, N2L 3G1 Canada

## Abstract

In zebrafish (*Danio rerio*), de novo synthesis of cortisol in response to stressor exposure commences only after hatch. Maternally deposited cortisol is present during embryogenesis, but a role for this steroid in early development is unclear. We tested the hypothesis that maternal cortisol is essential for the proper development of hypothalamus-pituitary-interrenal (HPI) axis activity and the onset of the stressor-induced cortisol response in larval zebrafish. In this study, zygotic cortisol content was manipulated by microinjecting antibody to sequester this steroid, thereby making it unavailable during embryogenesis. This was compared with embryos containing excess cortisol by microinjection of exogenous steroid. The resulting larval phenotypes revealed distinct treatment effects, including deformed mesoderm structures when maternal cortisol was unavailable and cardiac edema after excess cortisol. Maternal cortisol unavailability heightened the cortisol stress response in post-hatch larvae, whereas excess cortisol abolished the stressor-mediated cortisol elevation. This contrasting hormonal response corresponded with altered expression of key HPI axis genes, including *crf, 11B hydroxylase, pomca*, and *star*, which were upregulated in response to reduced cortisol availability and downregulated when embryos had excess cortisol. These findings for the first time underscore a critical role for maternally deposited cortisol in programming HPI axis development and function in zebrafish.

In response to stressors, the hypothalamus-pituitary-interrenal (HPI) axis responds in a coordinated manner leading to the release of cortisol into the circulation in teleosts^1^. The hypothalamus is the site of initial stressor recognition, resulting in the release of corticotropin-releasing factor (CRF)^2^,^3^, which acts on the anterior pituitary gland to synthesize and release adrenocorticotrophic hormone (ACTH) into the circulation. ACTH is produced by posttranslational modification of protein encoded by the gene proopiomelanocortin (*pomc*)^1^,^3^ ACTH binds to the melanocortin 2 receptor (MC2R) on the steroidogenic cells of the interrenal tissue and activates cortisol biosynthesis and secretion^3^,^4^. The interrenal tissue is analogous to the adrenal gland in higher vertebrates^5^ and cortisol is the primary circulating glucocorticoid in fish. Cortisol has a variety of effects in teleosts, but its most well-studied function in response to stress is to enhance metabolic capacity and mobilize energy stores to restore homeostasis^3^.

Recently, studies have described a role for glucocorticoid signaling in zebrafish development^4^. Glucocorticoid receptor (GR) transcripts are maternally deposited, but there is a complete turnover of these transcripts just after the mid-blastula transition and zygotically produced *gr* mRNA and protein are widespread in the embryo by 12–24 hpf^6^. Knockdown of GR translation in the newly-fertilized zebrafish embryo disrupts multiple developmental processes, including somitogenesis, myogenesis, and reduced overall growth and survival^6^,^7^. Also, exogenous cortisol administration impairs heart formation and downregulates key genes involved in cardiac development^8^. Other studies have also linked larval craniofacial development and osmoregulation to glucocorticoid signaling in the zebrafish embryos and larvae^9^,^10^. Altogether, these findings position GR as a major transcription factor mediating developmental programming in zebrafish^4^. While the effect of exogenous cortisol administration, as well as GR knockdown, on developmental phenotype have been explored, little is known about the contribution of maternal cortisol towards programming early development.

Maternal cortisol is deposited into the zebrafish oocyte prior to spawning and fertilization, and this steroid persists albeit in diminishing quantities during the course of embryogenesis. The *de novo* cortisol synthesis and activation of the HPI axis in response to stressors commences only after hatching^6^,^11^. Although a functional HPI axis response to stressor exposure is evident only after hatch, zebrafish do have all the molecular components for steroid biosynthesis and cortisol action prior to hatching^4^. In the developing hypothalamus, CRF-producing neurons are fully developed by 36 hpf, and *crf* is detectable from fertilization onwards^2^,^12^. The ACTH-producing corticotropes of the anterior pituitary are differentiated by ~26 hpf^5^ and although the interrenal tissue is not developmentally mature until post-hatching, it is the chromaffin cells and not the steroidogenic cells that are late to form and migrate to their final position^4^. Also, key steroidogenic enzymes, including 11ß-hydroxylase and steroidogenic acute regulatory protein (*StAR*), are expressed in the embryo by ~28 hpf^4^,^11^. Consequently, the stress hyporesponsive period during embryogenesis is thought to be critical for proper developmental programming^4^, but the possibility that maternal cortisol may play a role in HPI axis maturation or post-hatch function has not yet been tested.

Studies have linked neonatal stress or diminished maternal care with long-term programming changes to glucocorticoid stress axis activation^13^,^14^,^15^. Additionally, there is established evidence from mammalian models that levels of circulating glucocorticoids during development can affect the functioning of the stress axis in mature animals, including rats, guinea pigs, and sheep^16^,^17^. Also, studies in bird and reptiles suggest that maternal deposition of glucocorticoids into the yolk of eggs serve as a regulatory signal during development to prepare offspring for potentially stressful conditions^18^,^19^. Against this background, we hypothesized that maternally deposited cortisol is a key signal for post-hatch HPI axis development and function in zebrafish. To test this, we microinjected a commercially available cortisol antibody to sequester cortisol in the yolk of embryos, thereby reducing maternal cortisol bioavailability during early development. In parallel, we also microinjected cortisol into the yolk of embryos to artificially elevate cortisol content in the developing embryos. By examining the mRNA abundance of key genes involved in HPI axis functioning, as well as assessing the larval stress response, this study for the first time links maternal cortisol deposition as essential for stress axis programming in zebrafish.

## Results

### Confirmation of treatment efficacy

**Figure 1:**
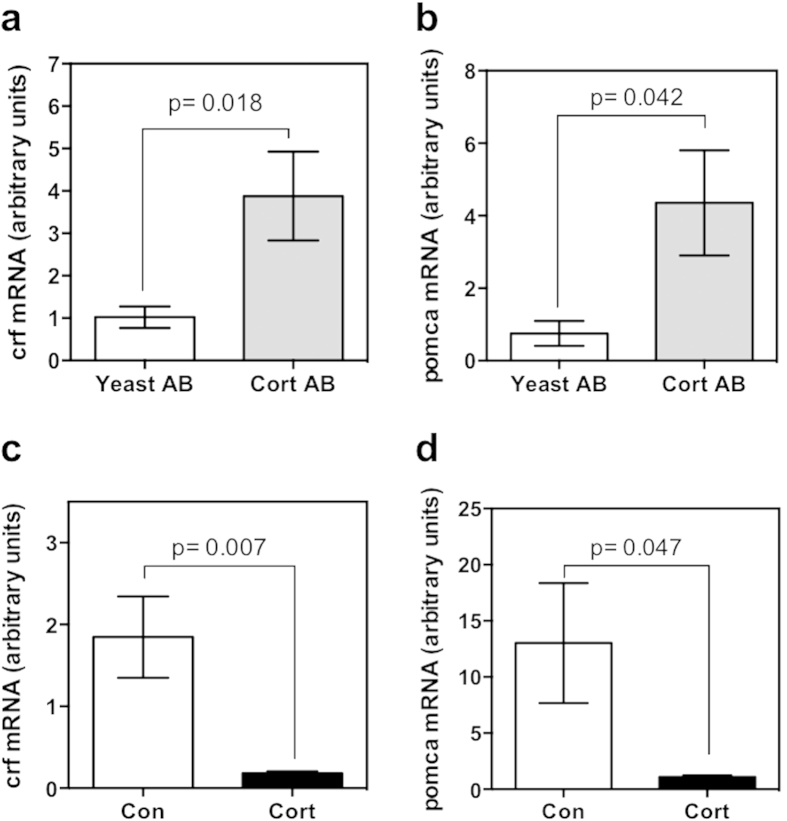
Modulation of maternal cortisol bioavailability. Expression of cortisol-responsive genes was quantified at 48 hpf as a measure of cortisol availability during embryogenesis. Transcript abundance of *crf* (**a**) and *pomca* (**b**) were significantly higher in the cortisol antibody (Cort AB) injected group compared to the yeast antibody (Yeast AB) controls. In contrast, microinjection of cortisol (Cort) significantly reduced *crf* (**c**) and *pomca* (**d**) mRNA abundance compared to the control group (Con). All data presented is mean ± SEM; *indicates significant differences (n = 6; each pool of 25 embryos; students t-test, p < 0.05).

To ensure that the injected cortisol antibody was able to sequester maternal cortisol effectively, expression of known cortisol-responsive genes were measured as a method of assessing cortisol availability during embryogenesis. Both CRF and POMC are known to be negatively regulated by cortisol via GR signaling as part of HPI axis feedback regulation^20^,^21^. Functional reduction in cortisol signaling by antibody sequestration of cortisol was confirmed by measurement of increased mRNA abundance of *crf* (Fig. 1a) and *pomca* (Fig. 1b) at 48hpf versus yeast-antibody injected controls. Additionally, increased zygotic cortisol content resulted in reduced mRNA abundance of *crf* (Fig. 1c) and *pomca* (Fig. 1d).

### Observation and characterization of embryo phenotypes

**Figure 2:**
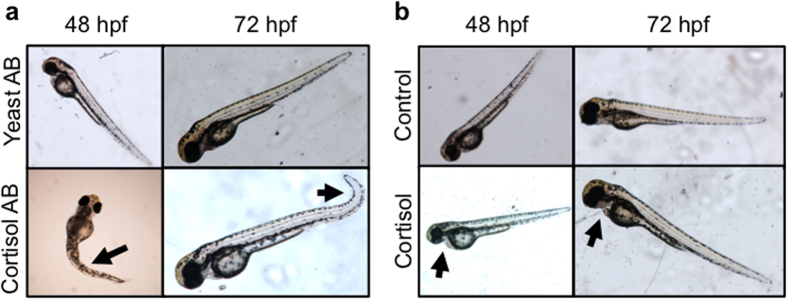
Maternal cortisol availability affects embryo phenotype. Embryos injected with cortisol antibody (Cort AB) or yeast-specific antibody (Yeast AB) (**a**) or with 32 pg of cortisol (Cort) or vehicle control (Con) (**b**), were imaged at 48 hpf (left images) and 72 hpf (right images). Representative images are presented. Cortisol-injected embryos displayed mild cardiac edema (see arrows), and cortisol antibody embryos displayed kinked tails and other disruptions in mesoderm formation (see arrows).

**Figure 3:**
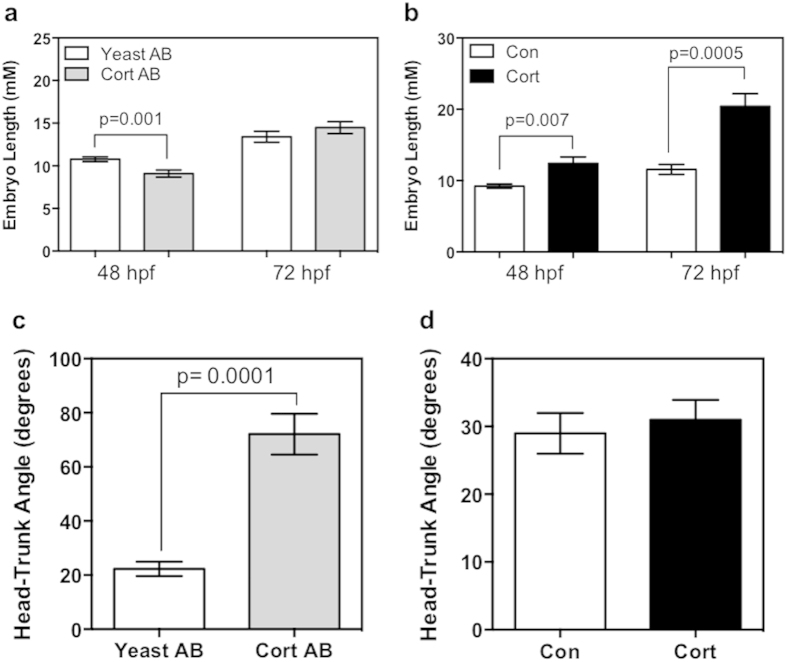
Maternal cortisol availability affects early development. Embryo growth was measured by the length from the tip of the head to the tail at 48 hpf and 72 hpf hpf for embryos treated with cortisol antibody (Cortisol AB) or yeast-specific antibody (Yeast AB) (**a**) to reduce available embryo cortisol content, and embryos injected with 32 pg of cortisol or vehicle control (**b**) to upregulate cortisol signaling in development. Cortisol upregulation resulted in increased embryo length at both time-points (**b**), while decreased cortisol signaling reduced embryo length only at 48 hpf (**a**). Embryo extension was measured by determining the angle between the ear-eye axis and the notochord in developing embryos at 72 hpf after treatment with either with cortisol or yeast-specific antibody injection (**c**), or 32 pg of cortisol or vehicle only control (**d**). Cortisol injection did not result in any change relative to control, but cortisol antibody injected embryos were less extended (indicated by a higher angle which represents a curled embryo). All data presented is mean ± SEM; *indicates significant differences (n = 6-7 embryos; students t-test, p < 0.05).

Embryos were observed for gross changes in morphology, and growth metrics were measured. The controls used in the two experiments, the vehicle-only (for cortisol-injection) and the yeast-specific antibody (for cortisol antibody-injection), showed no morphological differences at either 48 or 72 hpf (Fig. 2a,b), and were indistinguishable from the wild-type embryos. Morphological changes were observed with cortisol antibody injection, including moderate kinking of the tail and deformed curvature (see arrows; Fig. 2a). The images presented for the cortisol-injected embryos (see arrows; Fig. 2b) are similar to those seen in the “mild” or “unaffected” phenotypes seen before with cortisol injection, including only mild cardiac edema and no other major deformations^8^. In cortisol-antibody injected embryos, there was a decrease in embryo length (Fig. 3a) at 48 hpf (19% reduction), but no significant change was observed at 72 hpf. At 48 hpf, there was a significant increase in embryo length (Fig. 3b) in cortisol-injected embryos relative to controls (24% increase), and this change was even more pronounced at 72 hpf (76% increase). In addition to length, the angle between the eye-ear axis and the notochord was measured to assess embryo extension at 72 hpf. Cortisol-antibody injected embryos had a significantly increased angle relative to yeast-antibody treated embryos (Fig. 3c), displaying a pronounced curvature (see Fig. 2a) indicating a delay in embryo extension, but there was no change observed in cortisol-injected embryos relative to controls (Fig. 3d).

### Embryo cortisol concentration throughout development

**Figure 4:**
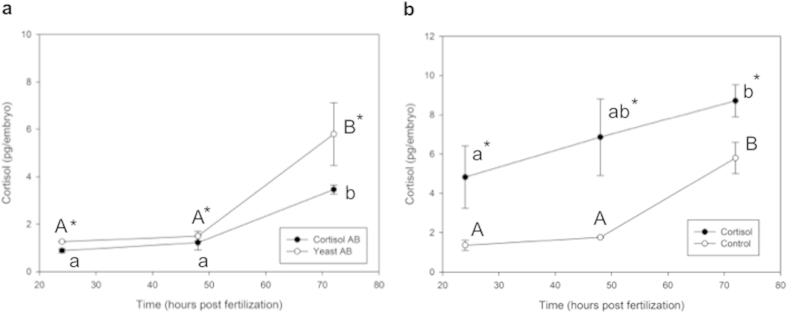
Embryo cortisol content during development. ELISA was used to measure cortisol in the developing embryos at 24, 48, and 72 hpf after treatment with either cortisol antibody (Cortisol AB) or yeast-specific antibody (Yeast AB) injection (**a**), or with 32 pg of cortisol or vehicle only control (**b**). Cortisol-antibody treatment significantly reduced cortisol content relative to yeast-antibody controls, while cortisol injection resulted in elevated cortisol content at all time-points relative to control injection. Both cortisol and cortisol-antibody treatments resulted in significantly elevated cortisol content at 72 hpf compared to their respective 24 hpf concentrations. All data presented is mean ± SEM; *indicates significant differences between treatments, while different same-case letters indicate significant differences between time-points for a specific treatment (n = 4; each pool of 15–25 embryos; two-way ANOVA, p < 0.05).

Cortisol levels in control embryos from both the exogenous cortisol treatment and the antibody treatment studies are consistent with previously published results^8^,^11^. Cortisol antibody treatment significantly reduced the available cortisol in the embryo compared to the control group (Fig. 4a). Two-way ANOVA revealed significant treatment (yeast AB versus cortisol AB; p = 0.001) and time (p = 0.001) effects, but no significant interaction between treatment and time (p = 0.500). Cortisol levels in the embryos were significantly higher at 72 hpf compared to the 24 and 48 hpf in both groups (Fig. 4a). Exogenous administration of 32 pg of cortisol into the newly-fertilized egg significantly elevated embryo cortisol concentration at all measured developmental time-points (Fig. 4b). The cortisol level at 24 hpf in the cortisol-injected group is only ~5 pg per embryo and we attribute this to a rapid efflux of the steroid after injection as previously shown^8^. Two-way ANOVA revealed significant treatment (control versus cortisol; p = 0.02) and time (p = 0.004) effects, but no significant interaction between treatment and time (p = 0.206). At 24 hpf, control-injected embryos had 1.35 pg/egg, compared to 4.83 pg/egg in cortisol-injected embryos. At 48 hpf, control-injected embryos remained relatively constant, containing 1.76 pg/egg, and cortisol-injected embryos did not show a significant increase either at 48 hpf (6.85 pg/egg). Only at 72 hpf was cortisol significantly elevated within each treatment group, with control-embryos containing 5.8 pg/egg and cortisol-injected embryos containing 8.71 pg/egg.

### Cortisol response to a physical stressor

**Figure 5:**
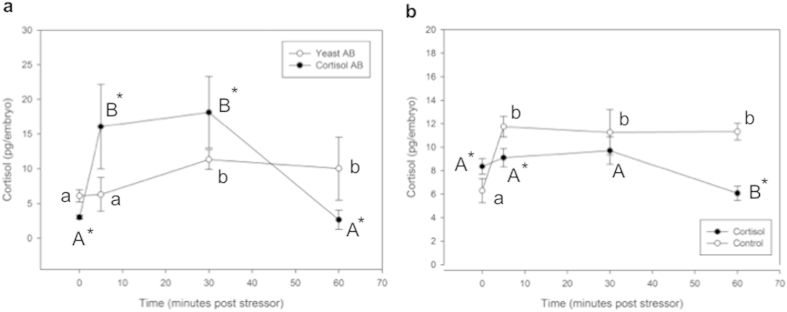
Cortisol response to a physical stressor. ELISA was used to measure changes in cortisol content during recovery from an acute physical stressor in embryos injected with either cortisol antibody (Cortisol AB) or yeast-specific antibody (Yeast AB) (**a**), or with 32 pg of cortisol or vehicle only control (**b**). Cortisol injection completely abolished the stress response relative to control embryos, with no observable rise in cortisol concentration at any time point, while control embryos showed a stressor-mediated cortisol response. Cortisol antibody injection accentuated but shortened the stress response in comparison to yeast-specific embryos, with higher cortisol at 5 and 30 minutes post stressor, but a reduced cortisol concentration after 60 minutes. All data presented is mean ± SEM; *indicates significant differences between treatments within a time-point, while different same-case letters indicate significant differences between time-points for a specific treatment (n = 6 except the Yeast AB at 60 min, which had only n = 3; each pool of 15 embryos; two-way ANOVA, p < 0.05).

The effects of treatments on the stress response were measured by quantifying 72 hpf larvae cortisol concentration during recovery from an acute physical stressor. A reduction in maternal cortisol bioavailability enhanced the stress response (Fig. 5a). Analysis by repeated measures two-way ANOVA revealed significant time effect (p = 0.002) and interaction of time and treatment (p = 0.02), but no significant effect of treatment alone (p = 0.338). Yeast-antibody injected control embryos had an initial cortisol concentration of 6.10 pg/egg, which was not significantly changed at 5 min post-stressor (6.29 pg/egg), but was elevated at 30 min (11.33 pg/egg) post-stressor and that level was maintained at 60 min (10.03 pg/egg) post-stressor exposure. Cortisol-antibody injected embryos had a significantly lower pre-stress cortisol level (3.02 pg/egg) than the yeast-antibody injected embryos, as was shown previously (Fig. 4a). After stressor exposure, cortisol levels increased significantly by 5 min (16.09 pg/egg) and remained elevated at 30 min (18.14 pg/egg) post-stressor exposure in the cortisol antibody injected group. At both of these time-points, cortisol-antibody injected embryos had significantly higher cortisol content than yeast-antibody injected embryos. At 60 min post-stressor, the cortisol concentration in the cortisol-antibody injected embryos had returned to pre-stress levels (2.66 pg/egg).

Cortisol-injected embryos displayed an abrogated stress response relative to control embryos (Fig. 5b). Analysis by repeated measures two-way ANOVA revealed significant time effect (p = 0.006) and interaction of time and treatment (p = 0.006), but no significant effect of treatment alone (p = 0.072). Control embryos had an initial cortisol concentration of 6.28 pg/egg, and showed a significant increase at 5 min post-stressor (11.74 pg/egg). Cortisol content in these embryos remained elevated and relatively constant at 30 (11.27 pg/egg) and 60 min (11.33 pg/egg) post-stressor exposure. Cortisol-injected embryos had a higher pre-stress cortisol level (8.36 pg/egg) and did not show a stress-induced increase in cortisol at either 5 (9.11 pg/egg) or 30 (9.70 pg/egg) min post-stressor, and in fact showed a decrease in cortisol at 60 min (6.08 pg/egg).

### Transcript levels of steroidogenic genes

**Figure 6:**
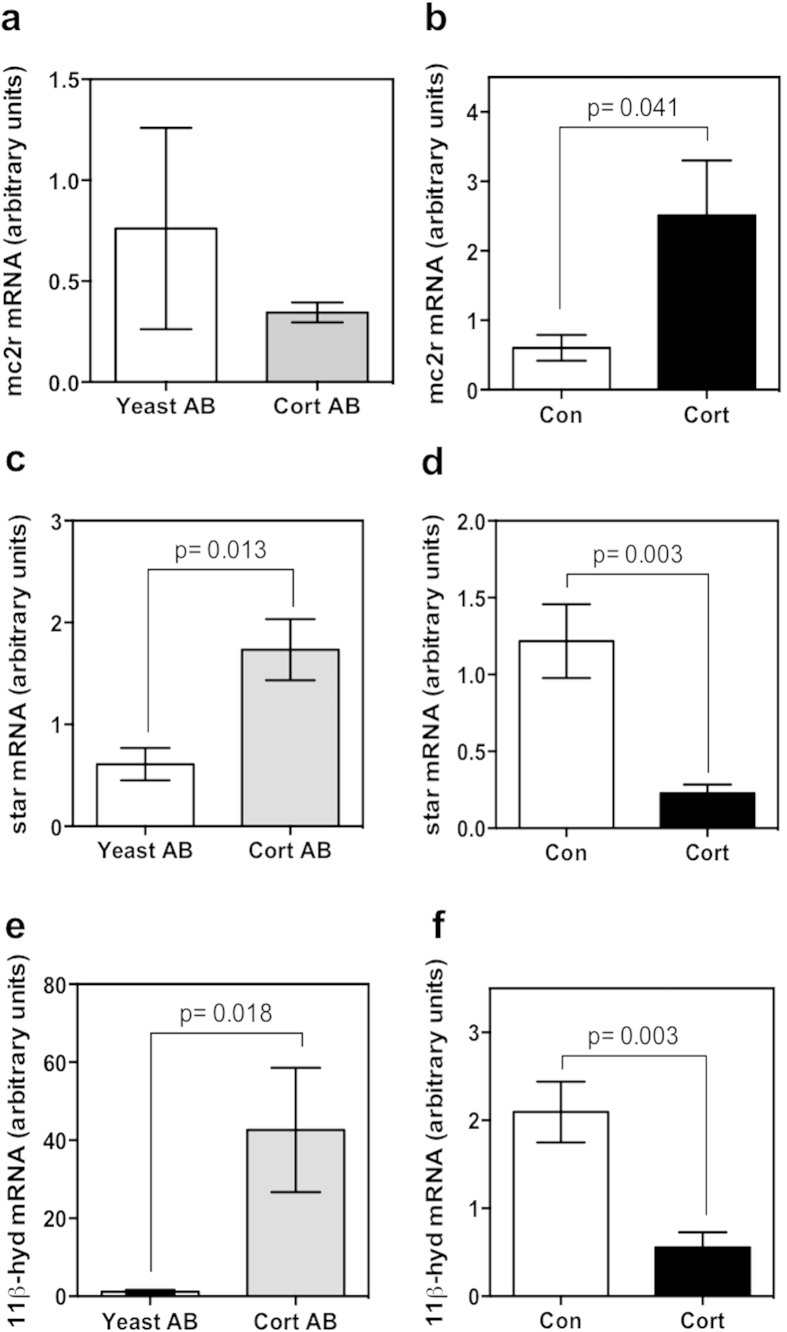
Steroidogenic gene transcript abundance. Quantitative PCR was used to measure the mRNA abundance of key genes involved in HPI axis function in 48 hpf embryos. Measured genes were: *mc2r* (**a,b**), *star* (**c,d**), and *11β-hydroxylase* (**e,f**) from embryos injected with either cortisol antibody (Cort AB) or yeast-specific antibody (Yeast AB) (**a,c,e**) or with 32 pg of cortisol (Cort) or vehicle only control (Con) (**b,d,f**). Expression of *11β-hydroxylase* and *star* were upregulated as a result of cortisol injection (relative to control embryos), and downregulated after cortisol antibody injection (relative to yeast antibody injected embryos), while expression of *mc2r* was upregulated only after cortisol injection. All data presented is mean ± SEM; *indicates significant differences (n = 6; each pool of 15 embryos; students t-test, p < 0.05).

In order to find mechanistic linkages for the disrupted stress response in treated embryos, mRNA abundance of key genes involved in steroidogenesis was measured by qPCR in 48 hpf embryos. In cortisol-antibody injected embryos at 48 hpf, the transcript levels of *mc2r* (Fig. 6a) did not change significantly, while *star* (Fig. 6c) and *11β-hydroxylase* (Fig. 6e) were significantly higher compared to the yeast-antibody injected control embryos. In the cortisol-injected embryos the transcript levels of *mc2r* (Fig. 6b) was significantly elevated, whereas the transcript abundances of *star* (Fig. 6d) and *11β-hydroxylase* (Fig. 6f) were significantly reduced compared to the controls.

### Transcript levels of corticosteroid receptor genes

**Figure 7:**
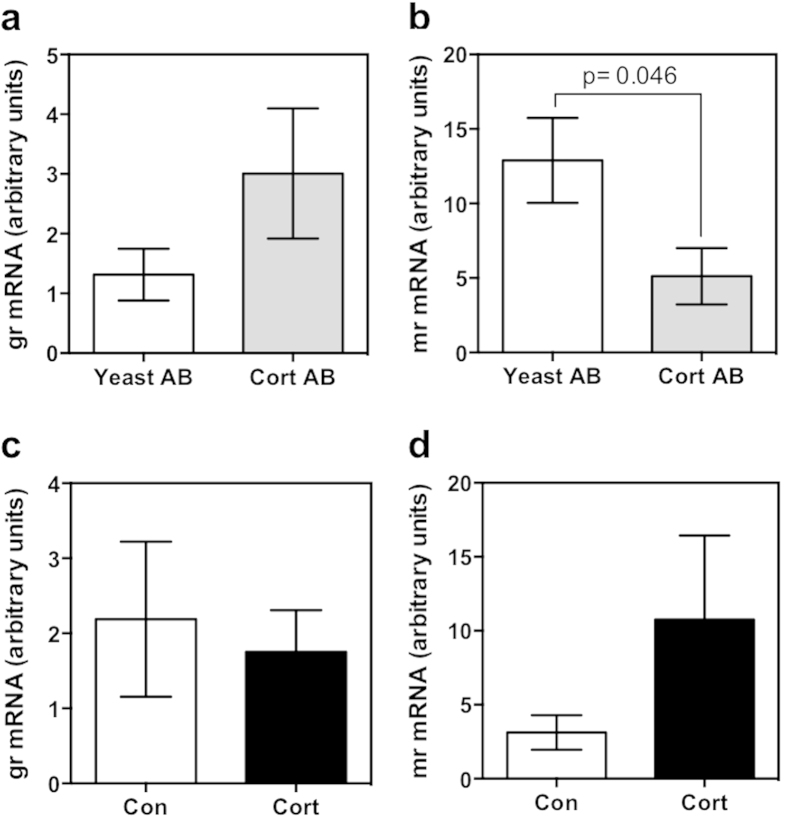
Corticosteroid receptor transcript abundance. Quantitative PCR was used to measure the mRNA abundance of corticosteroid receptors in 48 hpf embryos injected with either cortisol antibody (Cort AB) or yeast-specific antibody (Yeast AB) (**a,b**) or 32 pg of cortisol (Cort) or vehicle-only control (Con) (**c,d**). Expression of *gr* (**a,c**) was unchanged by either treatment. Expression of *mr* was significantly downregulated after cortisol antibody injection relative to yeast-antibody injected controls (**b**), but was unchanged by cortisol injection (**d**). All data presented is mean ± SEM; *indicates significant differences (n = 6; each pool of 15 embryos; students t-test, p < 0.05).

Cortisol antibody injection did not significantly affect *gr* (Fig. 7a) mRNA abundance, but significantly reduced the mRNA abundance of *mr* (Fig. 7b) compared to the yeast-antibody injected controls. There were no significant changes in the transcript abundances of *gr* (Fig. 7c) or *mr* (Fig. 7d) in response to cortisol treatment.

## Discussion

We demonstrate that maternal cortisol availability in newly-fertilized embryos is essential for the proper functioning of the cortisol stress axis in zebrafish larvae. Specifically, maternally deposited cortisol content modulates the transcript abundance of key genes involved in HPI axis activity, suggesting a key role for this steroid in programming stress axis development and function in zebrafish.

Maternal cortisol deposition into oocytes and its subsequent reduction in the developing embryo has been well established in teleosts^8^,^11^. In zebrafish, cortisol content in the newly-fertilized zygote is ~4 pg/embryo and this level decreases during embryogenesis until *de novo* synthesis commences after hatch and the concentration rises^8^,^11^. Although GR knockdown studies point to a key role for this receptor activation in regulating early zebrafish development^6^,^7^, it is unclear whether these changes reflect maternal cortisol effects. To test this, in the present study, we manipulated zygotic cortisol content by injecting cortisol-antibody into single-cell embryos to sequester maternally deposited cortisol, thereby reducing maternal steroid bioavailability during embryogenesis. This resulted in an increased transcript abundance of genes, including *crf* and the ACTH precursor *pomca*, that are repressed in response to GR activation in fish^20^,^21^ confirming reduced maternal cortisol bioavailability.

Lowering maternal cortisol availability increased the frequency and severity of morphological defects, including kinked tails, reduced growth, and slowed straightening of the tail during embryogenesis. Interestingly these phenotypic changes correspond to that observed in GR morphant zebrafish embryos^6^,^7^, supporting a key role for maternal glucocorticoid signaling through GR activation in the regulation of mesoderm formation during zebrafish development. While the mechanism is unclear, a previous study showed that reduced glucocorticoid signaling (by knockdown of GR) altered the transcriptome after the mid-blastula transition, implicating maternal cortisol signaling in the degradation of maternal mRNA levels^7^. Also, we have shown that GR signaling is essential for the regulation of bone morphogenetic proteins, a key morphogen involved in muscle growth and organogenesis, supporting a role for maternal cortisol in programming early developmental events^4^,^6^,^22^.

In addition to the effect on growth phenotype, our results demonstrate for the first time that maternal cortisol is critical for proper development and function of HPI axis activity in zebrafish. It is well established that the coordinated HPI axis response to stress commences only after hatch in zebrafish^4^,^11^,^12^. Our results indicate that a reduction in maternal cortisol availability modifies the cortisol response after hatch in zebrafish. Specifically, the lower whole body cortisol content in the larvae after hatch, coupled with a heightened cortisol response to a stressor insult points to impaired HPI axis functioning. In particular, the stressor-induced cortisol response was rapid and at a much greater magnitude compared to the control group pointing to an enhanced sensitivity of the cortisol stress axis post-hatch. This is supported by the higher mRNA abundance of key genes involved in HPI axis regulation^11^,^23^,^24^, including *crh*, *pomca*, and *star*, with lower maternal cortisol availability. Taken together, our results underscore a key role for maternal cortisol in regulating HPI axis maturation and functioning, and the tight regulation of this steroid content in the zygote may be essential for maintaining the stress hyporesponsive period during embryogenesis in zebrafish^11^,^12^. Another notable finding was the reduced mineralocorticoid receptor (MR) mRNA level with reduced cortisol bioavailability. The transcript abundance of MR, unlike GR, increases during embryogenesis, and is thought to play an important role in zebrafish early development^11^. As MR signaling may also be involved in HPI axis regulation^25^,^26^, we hypothesize that maternal cortisol affect on HPI axis development may involve MR signaling, but this remains to be determined. Overall, our findings clearly indicate that maternal cortisol is essential for developmental programming of post-hatch HPI axis functioning in zebrafish.

The contrasting larval growth phenotype and post-hatch stress axis activation response seen with zygotic cortisol manipulation (low versus high) indicates that a tight regulation of maternal cortisol deposition is essential for proper functioning of the HPI axis post-hatch in zebrafish. The results from the present study support our previous report showing distinct larval phenotypic traits, including cardiac edema with embryo cortisol excess in zebrafish^8^. Also, the suppression of *crf* and *pomca* in the present study confirms increased cortisol stimulation.

A major finding from this study was that excess cortisol in the zygote impairs the HPI axis functioning in zebrafish larvae. Specifically, these larvae failed to elicit a cortisol response after an acute physical stressor suggesting perturbation in HPI axis activity. This is supported by the downregulation of key genes involved in HPI axis functioning, including *crf*, *pomca*, *star*, and *11β hydroxylase*, pointing to a decreased capacity for steroid biosynthesis with excess zygotic cortisol content. The lower transcript levels of *crf* and *pomca* reflect a reduced activation of interrenal steroidogenesis, while the downregulation of *star* and *11β hydroxylase* supports a reduction in cortisol biosynthetic capacity^3^. This is seen despite an upregulation of *mc2r* transcript levels, leading to the proposal that this transcript change may be a compensatory mechanism to offset the lower ACTH stimulation due to excess zygotic cortisol. This lack of HPI axis activation at a critical time during development, especially given that the larvae will commence feeding soon after, will compromise the growth and stress performances leading to reduced fitness. This suggests that maternal stress and the associated transfer of excess cortisol to the embryos will compromise the HPI axis functioning in zebrafish larvae. Excess cortisol did not affect the transcript levels of corticosteroid receptors and is in agreement with our previous study^8^, and the phenotype seen is distinct from that observed in GR morphants suggesting a mechanism of action independent of GR downregulation. It is important to note that given the use of whole body mRNA from embryos and larvae, we may be observing changes in gene expression not only limited to HPI axis. However, the strong coordinated regulation of multiple genes involved in the HPI axis functioning, along with the whole body steroid response, supports a role for zygotic cortisol in regulating stress axis maturation. Together these results underscore the tight regulation of maternally deposited cortisol as essential for the proper development of post-hatch stress axis function in zebrafish.

The concept of maternal stress hormone deposition as a phenotypic programming molecule for offspring has been suggested for birds and reptiles, where changes in yolk corticosterone concentration affect post-hatch animal development^19^,^27^,^28^,^29^,^30^. Also, maternal stress and the attendant rise in embryo cortisol content can have lasting effects on offspring phenotype^31^,^32^,^33^. Our findings that embryo cortisol levels can influence the development and functioning of the HPI axis post-hatch positions zebrafish as a useful model for study of prenatal stress effects on long-term fetal programming. The use of zebrafish, a highly advantageous model for both developmental^34^ and endocrine^35^ studies, may allow for novel mechanistic insights into the effect of *in utero* excess glucocorticoid exposure on fetal development in placental mammals. Clearly maternal cortisol dynamics and control mechanism regulating this steroid deposition into the oocyte is an area of zebrafish development where further exploration is required^36^.

In conclusion, our findings provide a linkage between early embryo cortisol content and the post-hatch function of the cortisol stress response, including altered expression of key genes involved in HPI axis functioning. Our results demonstrate that HPI axis development requires precise regulation of zygotic cortisol availability, with reduced cortisol resulting in a more pronounced larval stress response and higher HPI axis gene expression, while excess cortisol suppresses key HPI axis genes and abrogates the expected response to a physical stressor. To our knowledge this is the first report in teleosts linking maternally deposited cortisol levels to altered cortisol responses and stress axis function in larvae. These findings have important implications for long-term animal fitness, especially given the highly conserved and important role for cortisol in stress adaptation^3^,^21^.

## Methods

### Zebrafish care and breeding

Care and breeding of adult zebrafish was carried out exactly as described previously^6^. Adult zebrafish were purchased from a commercial wholesaler (DAP International, Mississauga, ON) and maintained at 28 °C on a 14:10 light-dark cycle in an AHAB recirculating system (Aquatic Habitats, Apopka, FL) in deionized water supplemented with Instant Ocean salt, and fed twice daily with dry pellets (Ziegler, Gardners, PA). Zebrafish care protocols were approved by the University of Waterloo Animal Care Committee in accordance with the Canadian Council for Animal Care guidelines.

### Treatment injections

Adult zebrafish were placed in breeding traps (Aquatic Habitats) within system tanks before the onset of the dark period. Single-cell zebrafish embryos were collected from breeding tanks within 30 min of light exposure, and cleaned in system water prior to injection. All injections were performed exactly as described previously using a nitrogen-powered microinjector (Narishige, Japan) to inject a 1 nL injection volume into the yolk of single-cell embryos^6^. The injection treatments were as follows: undiluted cortisol antibody (MP Biomedicals, Solon, OH), previously characterized and used for radioimmunoassay studies^37^, selected after trying different dilutions ranging from 1:2 to 1:10; undiluted commercially available yeast-specific antibody (polyclonal rabbit anti-yeast GCN4, 200 μg/mL; Santa Cruz Biotechnology, Santa Cruz, CA), selected after trying various dilutions and did not affect embryogenesis and the phenotype was indistinguishable from wild-type embryos; 32 pg cortisol per egg as described before^8^; sterile vehicle control . GCN4 is a yeast-specific transcriptional activator with no known homolog in zebrafish^38^. The vehicle control was prepared exactly as the cortisol solution (dissolving in ethanol, then evaporation and reconstitution in sterile water). In our previous study, we classified a series of increasingly severe phenotypes for zebrafish embryos injected with 32 pg of cortisol^8^. In this study, only embryos displaying a phenotype without major deformations (“unaffected” or “mild” in the previous study^8^; ~74.7 ± 3.1% (n = 12 clutches of eggs) of embryos examined in this study) were used for analyses. The injection volume for all treatments was ~1 nL, injected directly into the yolk of the fertilized embryo. We observed ~20.5 ± 1.9% mortality in injected embryos versus uninjected controls, but there was no significant difference in mortality between treatment groups. After injection, embryos were reared in embryo medium^39^ and incubated at 28.5 ^o^C, with fresh medium at 12 and 36 hpf, as described previously^6^. After incubation, pools of 25 (pre-hatch) or 15 (post-hatch) embryos were collected at 4 (sphere stage), 12 (~10 somites stage), 24 (Prim-5 stage), 48 (Long-pec stage), and 72 (protruding mouth stage) hpf, flash frozen on dry ice, then stored at −80 ^o^C for later analysis of cortisol concentrations and transcript levels of key genes involved in HPI axis function. Each experiment was repeated four or six times for a sample size of n = 4 or n = 6.

### Characterization of embryo phenotypes

Embryos from each treatment group were observed throughout development for changes in morphology. At 48 and 72 hpf, embryos were observed and bright-field imaged with an AZ-100 dissecting microscope and DS-R1 camera (Nikon, Melville, NY). These images were then used to measure morphometrics in the developing embryos by use of annotation capabilities in the NIS-Elements software package (Nikon). Embryo growth and extension differences between treatment groups were assessed by two morphometrics: whole embryo length, and head-trunk angle (the angle between the eye-ear axis and the notochord), which were measured exactly as described previously^6^.

### Stressor exposure

At 72 hpf, larvae from each treatment group were exposed to a physical stressor to assess the functioning of the HPI axis in response to changes in cortisol concentrations in the early embryo. The stress protocol was performed exactly as described previously^8^. Briefly, 100 larvae were placed in a 100 mL beaker containing 80 mL of embryo medium, then swirled with a plastic pipette once per second for 60 s. Embryos were then allowed to recover over a 60 min period. Pools of 15 larvae were collected prior to stressor exposure, and at 5, 30, and 60 min post-stressor exposure. Larvae were then flash-frozen on dry ice and stored at −80 ^o^C for subsequent analysis of cortisol content.

### Cortisol extraction and quantification

Cortisol was measured from treated embryos during embryogenesis (24, 48, 72 hpf), as well as from 72 hpf treated embryos that were exposed to a physical stressor (pre-stressor and 5, 30, 60 min post-stressor). Pools of 25 embryos (24 and 48 hpf) or 15 embryos (72 hpf) were homogenized briefly (15 s) with a PowerGen 125 homogenizer (Fisher Scientific, Ottawa, Canada) in 200 μL of 50 mM TRIS buffer with added protease inhibitor (Roche, Laval, Canada). Cortisol was then extracted from these embryos with diethyl ether with slight modification of the method described previously^11^. Briefly, 1 mL of ether was added to the homogenate, which was then frozen on dry ice to isolate the aqueous phase and allow for decanting. This process was repeated for a total of three extractions and the ether was evaporated off overnight at room temperature. Cortisol quantification was performed with a commercially available ELISA (Neogen Corp, Lexington, KY) which has been used and characterized previously^8^,^40^, according to the manufacturer’s protocols. Steroid samples were reconstituted in extraction buffer from the ELISA kit. The extraction efficiency was 83 ± 4.5%, but uncorrected data is presented in the figures. The intra-assay coefficient of variability was 4.33%, while the inter-assay coefficient of variability was 8.9%.

### Gene expression

Relative expression of genes involved in HPI axis function was measured by real-time quantitative PCR (qPCR) in embryos from each treatment group at 48 hpf, in order to assess changes to HPI axis capacity that resulted from modulated embryonic cortisol content. RNA was extracted via the Ribozol-chloroform extraction process (Amresco, Solon, OH) according to the manufacturer’s protocols. Extracted RNA was quantified with a Nanodrop Spectrophotometer (Thermo Scientific, Ottawa, Canada). First-strand synthesis of cDNA was carried out with the High Capacity cDNA Reverse Transcription Kit (Applied Biosystems, Carlsbad, CA) with 1 μg of total RNA per sample. qPCR analysis was performed exactly as described previously^6^, with samples run in triplicate in an iCycler iQ thermocycler using iQ SYBR green supermix as a fluorophore (both from Biorad, Hercules, CA) with the following conditions: 94 °C for 2 min, 40 cycles of 30 sec at 95 °C, and 30 sec at Tm, followed by 10 min at 72 °C. The 25-μl qPCR mixture contained: 0.75 μl of template, 12.5 μl of iQ SYBR green, 0.75 μl of 10 μM primers, and 11 μl of ribonuclease-free water (Qiagen, Mississauga, ON). The genes measured were: *crf*, *gr*, *mc2r*, *mr*, *pomca*, *star*, and *11β hydroxylase*. Primer pairs, annealing temperatures, amplicon size, and references for previous characterizations of primers (if applicable) are presented in Table 1. Relative gene expression was quantified as described previously^6^ using the ΔΔC_t_ method^41^ with β-actin as a housekeeping gene (values were similar across all samples and therefore used as the normalizing gene).

### Statistical analysis

Statistical measures were performed using the Sigmaplot software package (Systat Software, Chicago, IL). Comparisons were made between cortisol injected and control injected embryos and between cortisol-antibody injected and yeast-antibody injected (control) embryos. Data are presented as mean ± standard error of the mean (SEM). Statistical comparison of cortisol concentrations during embryogenesis (24, 48, 72 hpf) were assessed by two-way ANOVA (p < 0.05) followed by a Bonferonni *post-hoc* test. Cortisol concentrations after exposure to a physical stressor (0, 5, 30, 60 min post-stressor) were assessed by repeated measures two-way ANOVA (p < 0.05) followed by a Bonferroni *post-hoc* test. All other comparisons (embryo growth metrics; qPCR analysis) were carried out using a student’s *t*-test (p < 0.05). Where necessary, data were log-transformed to meet the assumptions of normality and equal variance, while non-transformed data are shown in the figures.

## Additional Information

**How to cite this article**: Nesan, D. and Vijayan, M. M. Maternal Cortisol Mediates Hypothalamus-Pituitary-Interrenal Axis Development in Zebrafish. *Sci. Rep*. **6**, 22582; doi: 10.1038/srep22582 (2016).

## Figures and Tables

**Table 1:** Primers for quantitative real-time PCR.

Gene	Forward primer (5′-3′)	Reverse Primer (5′-3′)	Tm (C)	Amplicon size (bp)	Reference
*crf*	caccgccgtatgaatgtaga	gaagtactcctcccccaagc	60	118	
*gr*	acagcttcttccagcctcag	ccggtgttctcctgtttgat	60	116	11
*11β hydrox*.	tgtgctgaaggtgattctcg	gctcatgcacattctgagga	60	115	11
*mc2r*	ctccgttctcccttcatctg	attgccggatcaataacagc	60	127	11
*mr*	cccattgaggaccaaatcac	agtagagcatttgggcgttg	60	106	11
*pomca*	gaagaggaatccgccgaaa	ccagtgggtttaaaggcatctc	60	107	41
*star*	tcaaattgtgtgctggcatt	ccaagtgctagctccaggtc	60	122	11
*β-actin*	gtccctgtatgcctctggt	aagtccagacggaggatg	60	120	6

Forward and reverse primer pair sequence, melting temperature (Tm), amplicon size (bp), and the applicable references for corticotropin releasing factor (*crf*), glucocorticoid receptor (*gr*), 11*β* hydroxylase (*11β hydrox*), melanocortin 2 receptor (*mc2r*), mineralocorticoid receptor (*mr*), proopiomelanocortin a (*pomca*), steroidogenic acute regulatory protein (*star*) and beta-actin *(β-actin*) used in the present study.
